# PACKMAN-Molecule: Python toolbox for structural bioinformatics

**DOI:** 10.1093/bioadv/vbac007

**Published:** 2022-02-09

**Authors:** Pranav M Khade, Robert L Jernigan

**Affiliations:** Bioinformatics and Computational Biology Program, Roy J. Carver Department of Biochemistry, Biophysics and Molecular Biology, Iowa State University, Ames, IA 50011, USA

## Abstract

PACKMAN-molecule is a Structural Bioinformatics toolbox in the form of an Application Programming Interface that contains several utilities that can be used for structural bioinformatics applications. It has already been used in several applications, and its added features and unique object hierarchy make it readily extensible, feature-rich and user-friendly. The tutorial for it is available at: https://py-packman.readthedocs.io/en/latest/tutorials/molecule.html

**Availability and implementation:**

PACKMAN-Molecule is freely available with an MIT license on GitHub at https://github.com/Pranavkhade/PACKMAN.

## 1 Introduction

**Fig. 1.**
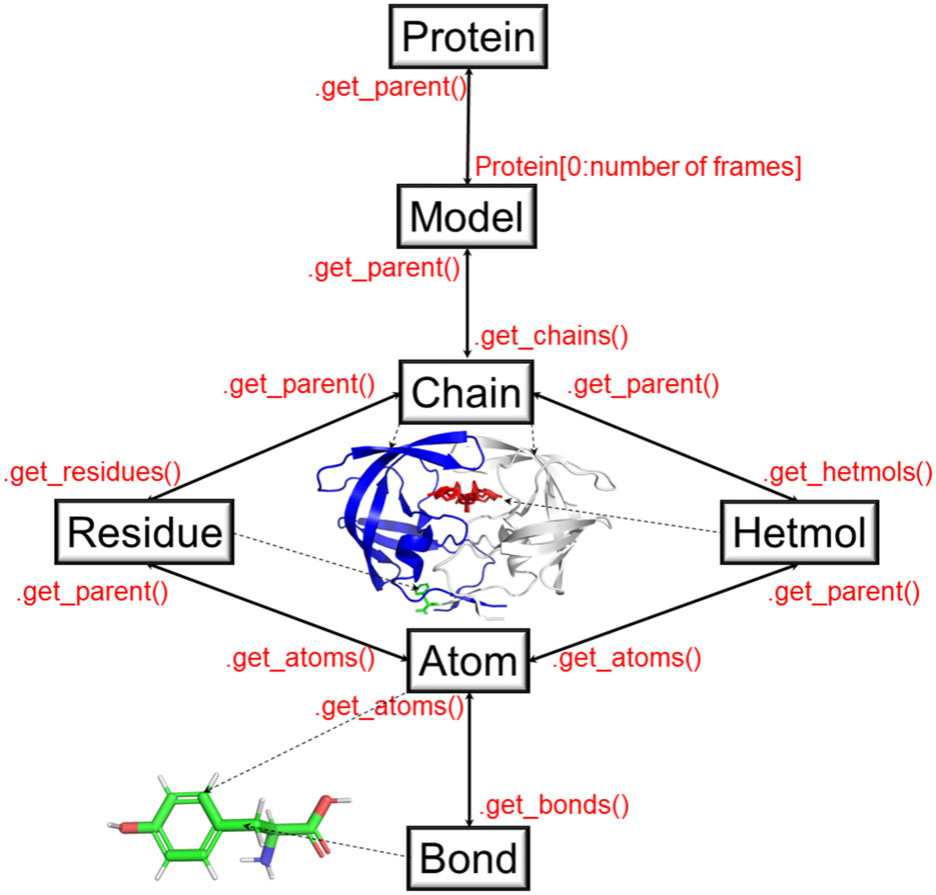
The object hierarchy of the PACKMAN-molecule submodule.

PACKMAN-Molecule is a submodule of a Python (www.python.org) package called PACKMAN that is freely available for major operating systems, i.e., for all that can run Python. The object hierarchy (Fig. 1) is the most critical feature of the package; it makes it easy to navigate molecular structures at different information levels to allow developers to institute different structural bioinformatics applications more quickly and efficiently. There are several application examples already published from the use of this submodule. The PACKMAN-Alpha Shape-Based Hinge Prediction (Khade *et al.*, 2020), PACKMAN-Compliance (Scaramozzino *et al.*, 2020) and hdANM (Khade *et al.*, 2021) are three examples.

Because of its robust array programming, the numerical aspects of all the objects are managed with NumPy (Harris *et al.*, 2020). At this time, PACKMAN already has nearly 27k downloads, and the userbase is expected to grow with the addition of utilities as more publications appear.

## 2 PACKMAN-Molecule utility and usage

The ‘Protein’ object is the highest object in the PACKMAN-Molecule hierarchy (Fig. 1); it contains all the information about a particular molecule. Even if the molecule is not an actual protein, it still is built around this ‘protein’ object. This is because the package is mainly for reading, writing and manipulating protein structures. However, any type of molecule, including small molecules, can be loaded using PACKMAN-Molecule. If a particular molecule information file has multiple frames (such as the Nuclear Magnetic Resonance experiments reporting multiple structures of the same protein), the ‘Protein’ object holds all of those frames as a ‘Model’ object in itself. Please visit the tutorial page (https://py-packman.readthedocs.io/en/latest/tutorials/molecule.html#tutorials-molecule). The detailed examples provide information on how to download and navigate a Protein, usually in mmCIF (Khade *et al.*, 2020; Westbrook and Bourne, 2000) or PDB file format from the RCSB Protein Data Bank website (Berman, 2000). Using the tutorial, the user can start with the ‘Protein’ object and navigate through the ‘Model’, ‘Chain’, ‘Residue’ and ‘Atom’ objects.

After learning to navigate, the programmer can interact with the object by using three principal functions (i) get functions, (ii) set functions and (iii) calculate functions. The get functions are used to get the features or sub-objects; for example, to get all the atoms of a residue, we can use the .get_atoms() function. Please note that get_atoms() is also available in the ‘Chain’ and the ‘Model’ objects to get all the atoms in respective objects. Similarly, another example of the get object is the get_location() function of the ‘Atom’ object that gives the user a NumPy array of length three having x, y and z coordinates of the atom in space. The set methods are used when the user wants to manipulate an object or assign properties to the objects. For example, the set_location([x, y, z]) function for the ‘Atom’ object with an array of length three as an argument would set/change a particular atom's location. The calculate functions work as the name suggests. These perform some calculations in the background and return the result to the user. For example, the calculate_distance() function of the ‘Atom’ object with another ‘Atom’ object as an input will provide the Euclidean distance between two atoms.

It is important to note that many new features will be added to this package as this is a continually improving package. However, the basic structure for navigation and interactions (get, set and calculate methods) makes the package user-friendly and are universal throughout the package. There are few exceptions to this pattern of ‘get’, ‘set’ and ‘calculate’ methods in various library objects, such as the write_structure() function of the ‘Proein’ object. However, such functions are obvious and can be looked up using the PACKMAN-Molecule module page for the respective object (https://py-packman.readthedocs.io/en/latest/py-modindex.html). 

## Funding

This work was supported by the National Institutes of Health (NSF) [DBI-1661391 and NIH R01GM127701].

*Conflict of Interest*: none declared.
